# A Fast Semiautomatic Algorithm for Centerline-Based Vocal Tract Segmentation

**DOI:** 10.1155/2015/906356

**Published:** 2015-10-18

**Authors:** Anton A. Poznyakovskiy, Alexander Mainka, Ivan Platzek, Dirk Mürbe

**Affiliations:** ^1^Department of Otorhinolaryngology, University Hospital Carl Gustav Carus, Technische Universität Dresden, 01062 Dresden, Germany; ^2^Division of Phoniatrics and Audiology, Department of Otorhinolaryngology, University Hospital Carl Gustav Carus, Technische Universität Dresden, 01062 Dresden, Germany; ^3^Voice Research Laboratory, Hochschule für Musik Carl Maria von Weber, 01067 Dresden, Germany; ^4^Department of Radiology, University Hospital Carl Gustav Carus, Technische Universität Dresden, 01062 Dresden, Germany

## Abstract

Vocal tract morphology is an important factor in voice production. Its analysis has potential implications for educational matters as well as medical issues like voice therapy. The knowledge of the complex adjustments in the spatial geometry of the vocal tract during phonation is still limited. For a major part, this is due to difficulties in acquiring geometry data of the vocal tract in the process of voice production. In this study, a centerline-based segmentation method using active contours was introduced to extract the geometry data of the vocal tract obtained with MRI during sustained vowel phonation. The applied semiautomatic algorithm was found to be time- and interaction-efficient and allowed performing various three-dimensional measurements on the resulting model. The method is suitable for an improved detailed analysis of the vocal tract morphology during speech or singing which might give some insights into the underlying mechanical processes.

## 1. Introduction

The process of human voice production involves a complex interaction of different components and mechanisms. It involves the generation of a pulsating transglottal airflow which is filtered by the vocal tract (VT) resonator. The shape of the VT, the aeroacoustic cavity between the vocal folds and the lips, defines the formant frequencies and the frequency response of the filter which, in turn, defines vowels, consonants, and essential parts of voice timbre [1].

Magnetic resonance imaging (MRI) has become a promising technique for investigating the VT at a functional stage. MRI delivers images of high spatial resolution which can be analyzed in two dimensions within a single sagittal plane allowing for detailed analysis of dynamic VT adjustments during speech or even singing. For a detailed overview, see [2].

The elongated structure of the VT along with its curved shape makes segmentation feasible with the use of centerline-based methods. In medical imaging, these approaches have been successfully used in vessel segmentation [3]. The image stack is transformed into a coordinate system which is aligned with the centerline, and the cross sections are segmented. This makes a reduction of the 3D segmentation problem to a set of two-dimensional problems possible.

In many cases, the estimation of the centerline is a problem in itself, as in the aforementioned vessel segmentation task. There exist a broad variety of segmentation methods which compute the centerline on runtime, based on segmentation results of previous cross sections. Some of them postulate a circular [4] or else analytically defined [5] cross-section shape. Other authors make use of deformable models, subsequently skeletonizing the shape and extrapolating the resulting centerline, such as Li and Ourselin [6]. In our recent study on the cochlea [7], we also used deformable models in form of active contours [8] but predicted the centerline via mass centers of cross sections with the help of a Kalman filter [9].

With regard to segmentation, the geometrical structure of the VT poses similar challenges as vessel or cochlear segmentation. The potential of tomographic imaging techniques, especially MRI, to deliver three-dimensional (3D) image stacks of the VT has been exploited, for instance, to analyze area functions during sustained phonation [10, 11]. So far though, applications to the VT mostly included manual segmentation or relatively simple segmentation algorithms. Although publications on 3D modeling of the VT exist for quite some time [12, 13], they rely on manual segmentation.

A few centerline-based methods have been applied to the VT. However, most studies focus solely on the extraction of the area function by employing 2D methods such as threshold segmentation [14, 15]. A 3D centerline-based approach was presented by Vampola et al. [16], yet it still suggests segmenting individual cross sections manually. While manual segmentation requires little implementation error and is thereby a good tool for exploratory analysis, it has the drawback of being time-consuming, a factor that precludes its application to large sets of individual data.

There are several studies published which use region growing for the segmentation of VT [17, 18]. This method does not allow for the immediate studying of VT cross sections as it lacks a centerline. In order to measure cross-sectional areas, the centerline has to be constructed a posteriori, and the resulting segmented body cut along this centerline. This process might be nontrivial if there are bifurcations along the path. Indeed, VT has minor bifurcations: the piriform sinuses (*sinus piriformes*) and* vallecula*. On the other hand, vocal tract cross-sectional area has attracted considerable interest within the research community [19–23], since it plays an important role in the acoustics of speech and singing. Functional VT adjustments during phonation seem to be of importance not only for educational purposes but also for the medical field, where voice problems, for example, among professional voice users continue to bring about considerable socioeconomic burdens for the health care systems [24, 25].

Thus, in this study, we attempt to develop a VT segmentation algorithm which satisfies the following: (1) reduced operator interaction and time efforts and (2) direct data output on both VT cross sections and 3D geometry.

## 2. Materials and Methods

### 2.1. Image Data Acquisition and Sound Recording

A 43-year-old male test subject (height: 1.90 m, weight: 108 kg) was asked to produce a sustained vowel in a 3.0-T MR system (Verio; Siemens Medical Solutions, Erlangen, Germany) and to keep articulation constant during the recording. The task was specified regarding vowel quality (closed midback rounded vowel /o/ as in German “Boot”), pitch (220 Hz/ A3), and phonatory condition (speaking voice). The MRI recording was initiated as soon as the subject had started phonation. The MRI was performed with a 12-element head-neck coil. The applied MRI sequence was a volumetric interpolated breath-hold examination sequence with an acquisition time of about 12 s. A set of 52 sagittal slices of the whole VT was obtained. The parameter setting was the following: slice thickness 1.8 mm, repetition time 4.01 ms, echo time 1.22 ms, matrix 288 × 288, field of view 300 × 300 mm, and flip angle 9°. The obtained resolution of the images was 1.04 mm. Due to the known limitations of the MRI to visualize structures with low water content, the teeth were not detected in the MRI scan. For the segmentation of the oral cavity the segments were forced manually to remain between the tongue and the maxillary bone leaving out the space of the teeth.

An optical microphone unit (MO 2000 from Sennheiser) and a laptop PC running Audacity software (Dominic Mazzoni et al., http://audacity.sourceforge.net/, retrieved on January 20, 2015) were used for sound recording within the MRI facility. The acoustical recording was used to ensure vowel quality and pitch correctness.

### 2.2. Processing of Images and Coordinate Transform

The 52 sagittal images were stacked and scaled by a factor of 3.0 with ImageJ (National Institutes of Health, Bethesda, MD, USA) resulting in 156 images with a pixel size of 0.35 mm. This scaling was necessary to facilitate the later segmentation. Then, the images were resliced to the coronal view in order to fit the distance between slices to 0.35 mm and to obtain uniformly sized voxels. The reslice was repeated a second time with default settings to obtain sagittally oriented images.

For further image processing, the used algorithms were implemented in our software IPTools (freeware: http://www.uniklinikum-dresden.de/das-klinikum/kliniken-polikliniken-institute/hno/forschung/forschungslabor-gehor/links, last inspected January 20, 2015). In order to increase the grayscale gradient at the air-tissue border of the VT, the image stacks were filtered using anisotropic diffusion [26].

**Figure 1:**
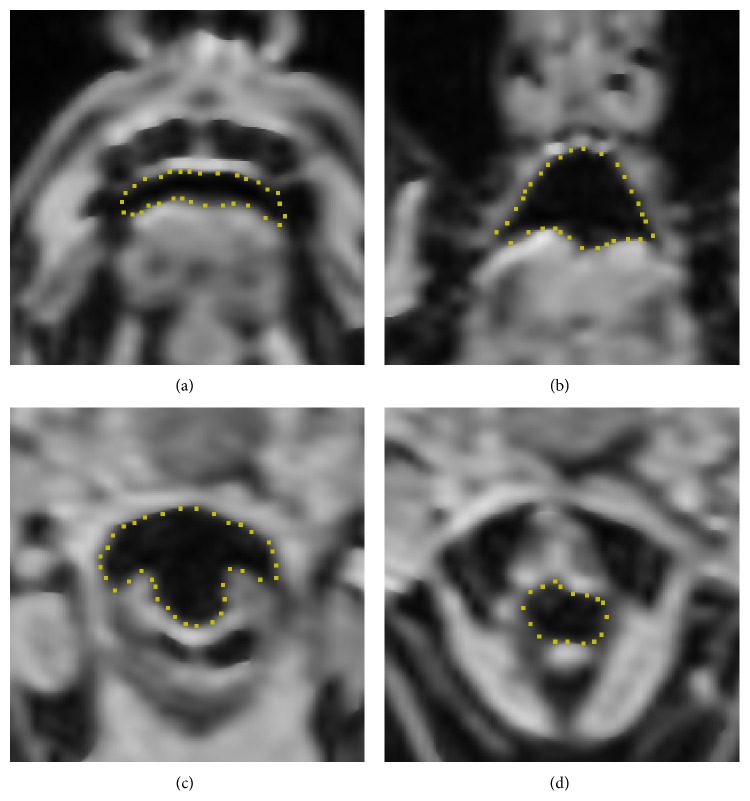
Four examples of vocal tract cross sections at different levels: (a) anterior oral cavity, (b) central oral cavity, (c) hypopharynx at inferior vallecula, and (d) larynx.

On the midsagittal image of the stack, the centerline was drawn (see Figure 1). This was done by defining node points such that the centerline intersects the tip of the uvula and the crossing of ventricular folds and the arytenoids. This procedure was established to ensure repeatability for application to other subjects while keeping the orientation of transformed slices near-orthogonal to the pharyngeal axis. Moreover, this provides a near-parallel slicing of the ventricular folds which is essential for calculating the area function in this region.

The centerline was piecewise interpolated between these nodes using cubic splines:

(1) $$\begin{array}{c} c_{i}\left(\;t_{c}\;\right)=a_{i,3}t^{3}+a_{i,2}t^{2}+a_{i,1}t^{1}+a_{i,0}, \end{array}$$

with **c**_*i*_ = [*c*_*i*,*x*_, *c*_*i*,*y*_, *c*_*i*,*z*_]^T^ and *t*_*c*_ ∈ {0,…, 1}. The four coefficients were defined by setting the positions at *t* = 0 and *t* = 1 to the coordinates of the nodes and equalizing the first derivatives d**v**/d*t* at these positions with adjacent spline segments.

Subsequently, the image stack was transformed along the curve with a fixed spacing of 1 pixel between new images resulting in a distance of 1.04 millimeters between centers of images. The center of each image of the new stack was set at the respective position on **c**(*t*_*c*_). The coordinate axes were set to the Frenet vectors of the curve at this position; specifically, the *x*-axis was set to the normalized binormal vector **b**, the *y*-axis to the negated normalized normal vector −**n**, and the *z*-axis to the normalized tangent vector **t**. The negation of the normal vector was necessary since the coordinate system of an image stack was defined as left-hand.

From these definitions, we could derive the following affine transform matrix for each *t*_*c*_:

(2) $$\begin{array}{c} T=\left[\begin{array}{cccc} b_{x} & b_{y} & b_{z} & c_{x}\\ {\ddot{-c}}_{x} & {\ddot{-c}}_{y} & {\ddot{-c}}_{y} & c_{y}\\ {\dot{c}}_{x} & {\dot{c}}_{y} & {\dot{c}}_{z} & c_{z}\\ 0 & 0 & 0 & 1 \end{array}\right]. \end{array}$$

Note that the binormal vector **b** = [*b*_*x*_, *b*_*y*_, *b*_*z*_]^T^ is defined as

(3) $$\begin{array}{c} b=\dot{c}\times \ddot{c}. \end{array}$$

The transformation with a set of such matrices delivered a stack of several hundred images, where each image displayed the cross section in a manner feasible for 2D segmentation.

### 2.3. Segmentation

The segmentation was performed with a greedy variant of active contours [8]. A circular discrete starting contour was initialized with a center at user-defined position. Each node of the contour had the energy balance

(4) $$\begin{array}{c} E=E_{\text{cont}}+E_{\text{curv}}+E_{\mathrm{ext}}+E_{\text{dev}}. \end{array}$$

Here, *E*_cont_ is the contour energy which controls the expansive behavior of the contour. It is defined by a first-order derivative of the active contour curve function **v**(*t*_*v*_):

(5) $$\begin{array}{c} E_{\text{cont}}=\alpha \cdot {\left|\;\nabla v\left(\;t_{v}\;\right)\;\right|}^{2}. \end{array}$$

*E*_curv_ is the curvature energy which models the bending stiffness of the contour via the second-order derivative of the curve function:

(6) $$\begin{array}{c} E_{\text{curv}}=\beta \cdot {\left|\;\Delta v\left(\;t_{v}\;\right)\;\right|}^{2}. \end{array}$$

The external energy *E*_*ext*_ provides the contour with edge detection and is proportional to the negated square norm of the grayscale gradient:

(7) $$\begin{array}{c} E_{\mathrm{ext}}=-\gamma \cdot {\left|\;\nabla I\left(\;x,y\;\right)\;\right|}^{2}. \end{array}$$

Finally, the deviation energy *E*_dev_ provides cross links between contours on adjacent tomogram images:

(8) $$\begin{array}{c} E_{\text{dev}}=\delta \cdot d^{4}. \end{array}$$

Here, *d* is the distance to the nearest node of the contour on previous image. In the first contour, this energy is set to zero. The Greek letters in (5)–(8) indicate user-defined parameters.

A search for the local minimum of the energy sum over all nodes was performed. This caused the contour to expand and adapt to the cross section of the vocal tract iteratively. When the contour stopped moving, the finding of local energy minimum was stated and the algorithm moved to the next image. Alternatively, the processing of the contour on an image stopped when the number of iterations exceeded a predefined threshold (*n* = 30). On the next image, a new contour was initialized with the end result of the previous image.

As the contour expanded, new points were added between any two neighboring points, whose spacing exceeded a predefined value *s*_max⁡_. Similarly, one of two points was deleted if the spacing became lower than *s*_min⁡_ after any iteration. For this purpose, the values were defined as *s*_max⁡_ = 4*s*_min⁡_ and *s*_min⁡_ = 3 px.

The algorithm progress through the image stack was terminated at user's command. The accuracy of the segments was checked by an experienced laryngologist and corrected manually if needed.

The resulting segment stack was realigned with the information of the centerline curvature and visualized using Amira (FEI Visualization Sciences Group, Burlington, MA, USA).

## 3. Results and Discussion

Using the abovementioned methods, we were able to segment cross sections along the entire vocal tract (Figure 1). Total time used for segmentation (excluding filtering with anisotropic diffusion) was about 90 minutes.

**Figure 2:**
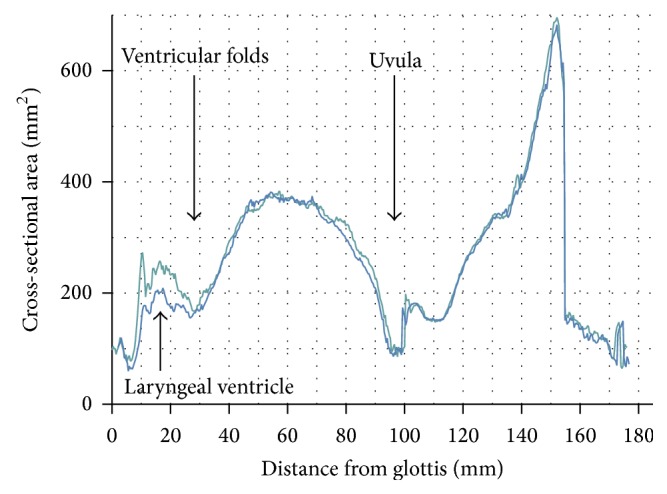
Cross-sectional area function obtained from two segmentations of the same data set.

To estimate algorithm objectivity, we performed the segmentation including centerline positioning twice. Out of the segmented data, we computed the area functions which are plotted against each other on Figure 2. The graphs appear to be highly correspondent down to the ventricular folds, scattering only in the region of the laryngeal ventricle. We assume that this is due to phonatory vibrations which cause blurring artifacts on VT borders and decrease the precision of segmentation.

We calculated reference volumes by superposition of segment areas. The total volume of the VT was estimated at 50528 mm^3^. The volume of the oral cavity was 25965 mm^3^, the combined volume of oropharynx and hypopharynx (segments from* uvula* to arytenoids including the* sinus piriformes*) was 21900 mm^3^, and the volume of larynx (segments from arytenoids to glottis) was 2663 mm^3^.

**Figure 3:**
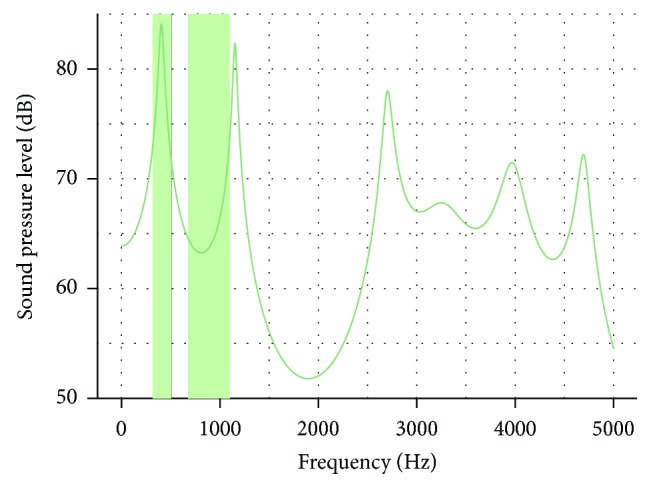
Transfer function computed with PRAAT based on the calculated area functions (Figure 2, green line). Light green stripes denote frequencies of the first two formants according to [27].

As a method of validation, we calculated the acoustic transfer function of the VT using PRAAT (Paul Boersma and David Weenink, http://www.fon.hum.uva.nl/praat/). It is displayed in Figure 3 and shows distinct peaks which are fairly coincident with the 1st and the 2nd formants for the utilized vowel [27].

**Figure 4:**
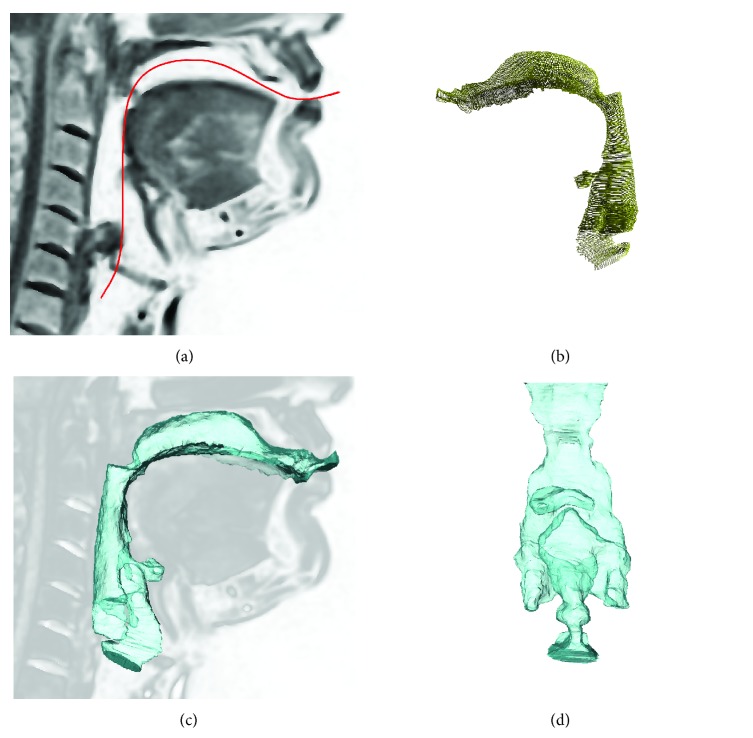
Vocal tract geometry modeling results: (a) centerline position within a midsagittal slice, (b) spatial alignment of the resulting cross-sectional segments, (c) generated surface mesh and its location within the image stack, and (d) detailed coronal view of the lower vocal tract.

With the obtained VT cross-section model, the geometrical analysis within all three spatial dimensions becomes feasible. The complete set of segmented cross sections, transformed from centerline-based coordinate system back to the global coordinate system (Figure 4(a)), is shown in Figure 4(b). Triangulation of this set yielded a surface mesh of the vocal tract shown on Figure 4(c). A close-up on the lower VT showing the high-detailed resolution of the larynx segmentation is displayed in Figure 4(d). This resulting mesh can serve as direct input for further numerical simulations using, for example, finite element modeling.

The accuracy of the model is dependent on the used MRI tomography device and the stability of the test subject over time. A natural challenge to the stability is the requirement to the subject to keep a constant articulatory setting and to maintain phonation during the entire recording procedure. This is necessary to produce enough images to cover the whole VT. There are hints that the movement artifacts of the jaw during sustained phonation are in the submillimeter order [28]. Yet this data represents only a single subject. A detailed discussion of the accuracy of MRI investigations prior to image processing is beyond the scope of this methodological study but ought to require further scientific attention.

The technical accuracy of the segmentation algorithm is constrained by the obtained resolution of MRI images. Since active contours cannot perform segmentation to a higher precision than 1 pixel, the uncertainty in border estimation corresponds to the resolution value, that is, 1.04 mm, which is well in range of state-of-the-art publications [15, 18]. Hence, the error in cross-section area estimation is between ca. 20 and 50 mm^2^, depending on the area value.

The amount of input image data calls for a time-efficient and at least semiautomatic algorithm in order to reduce man-hours spent on segmentation. Unlike vessels whose cross-section shape does not vary much along the centerline, the VT has highly variable cross-section geometry (cf. Figure 1). A heuristic algorithm which is capable of handling arbitrary shapes is active contours which are widely used in biological imaging [29, 30]. It is based on the search of a steady-state shape of discrete deformable contour under influence of an equilibrium of internal and external forces. Internal forces govern the intrinsic properties of the contour, expansion, and stiffness. The external force creates a link to the image data, attracting the contour to regions with the greatest gradient of grayscale intensity.

## 4. Conclusion

By the presented approach for a complete MRI based 3D segmentation of the VT at a functional state, we were able to obtain a high-detailed model. The method could be used for answering questions regarding the physics and mechanical properties of the VT.
